# Long term exposure to dietary cannabinoids inhibits rapid functional tolerance to ethanol in *Drosophila melanogaster* adults

**DOI:** 10.17912/micropub.biology.001825

**Published:** 2025-12-11

**Authors:** Alyssa Vidal, Sandra Illescas, Vivian Le, Nathan Feldhorn, Sarah Khan, Mariano Loza-Coll

**Affiliations:** 1 Biology, California State University, Northridge, Northridge, California, United States

## Abstract

In rodents, exposure to cannabidiol (CBD) was shown to alter responses to alcohol, primarily via CB1/CB2 signaling. However, CBD also targets non-canonical receptors whose potential roles in modulating CBD-alcohol interactions remain unknown. We used a simple behavioral assay to compare responses to ethanol in *Drosophila melanogaster* , which lacks CB1/CB2 genes but has orthologs for non-canonical targets. Flies fed with a CBD-enriched diet retain baseline ethanol sensitivity but fail to develop rapid functional tolerance to ethanol. These findings support using *Drosophila* as a model to study non-canonical CBD signaling in ethanol behaviors relevant to the development of alcohol use disorder.

**Figure 1. Sedation assay set up, data collection and analysis to determine how adult and larval exposure to dietary cannabinoids affect the development of rapid functional tolerance to ethanol (RFTE):**
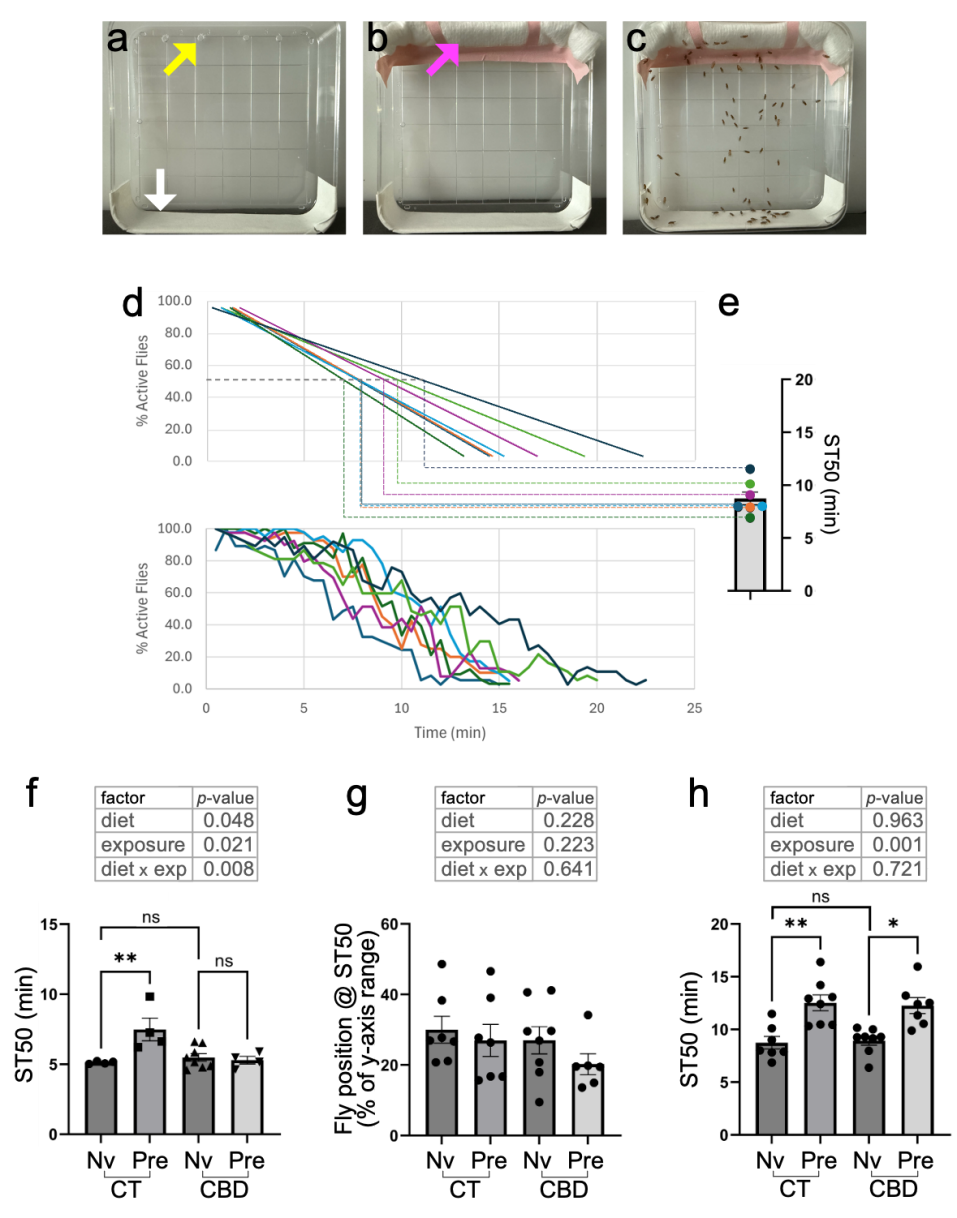
**(a)** Ethanol sedation cages are prepared by perforating four pinholes near the edge on the back side of a 10cm square Petri dish (yellow arrow), and affixing clear tape to the lateral side on the opposite end (white arrow). This needs to be done only once. **(b)** Prior to each sedation assay, a 10cm-wide gauze rod is secured with tape to the top of the cage, covering the pinholes (magenta arrow). **(c)** Example of a picture with flies being exposed to ethanol vapors by soaking the gauze rod with 4mL of ethanol and sealing the cage. All non-sedated flies across pictures were spotted using the multi-selection tool in ImageJ/Fiji, and Y-coordinate values were used to calculate average %Active flies and %Y-climb (as explained in Methods). **(d)** Raw line plots of %Active flies over time (bottom), and their corresponding linear regressions (top). A standard line equation was used to calculate ST50 values, i.e., the time at which 50% of the flies in a group are projected to become sedated. **(e)** ST50 values from each student group were pooled as biological replicates for each sample in subsequent statistical analyses. **(f)** ST50 values of adult flies on a 14-days control (CT) or cannabinoid (CBD) diet, and exposed to ethanol vapors for the first time (naïve, Nv) or following a prior intoxication, 4 hrs earlier (Pre-exposed, Pre). **(g)** Average position of flies along the y-axis (expressed as % of y-axis range) at their corresponding ST50 times. **(h)** Comparison of ST50 values across groups as in (f), except that the flies were exposed to cannabinoids (or not) as larvae, not as adults. The top tables in (f) thru (h) show the outcomes of 2-way ANOVAs, testing data across diet (CT or CBD), exposure (Nv or Pre) and their interaction. It should be noted, however, that not all samples passed a Shapiro-Wilk’s test of normality.

## Description

There has been increased interest in the potential health benefits of cannabidiol (CBD) and other non-psychotropic cannabinoids, particularly in the context of their anti-inflammatory, neuroprotective and analgesic effects (Bhunia et al., 2022; Cásedas et al., 2024; Voicu et al., 2023). In mammals, cannabinoids are thought to function primarily via their cognate G protein-coupled receptors CB1 and CB2 (reviewed in Shabazi et al., 2020 and Coelho et al., 2023). However, cannabinoids have also been shown to exert a broad range of physiological and neurological phenotypes via so-called non-canonical molecular targets, such as TRPV1-4 and TRPA1 (Etemad et al., 2022), GPR55 (Rosenberg et al., 2023), PPARγ (Khosropoor et al., 2023; Puighermanal et al., 2024), the 5-HT1A serotonin receptor (Linge et al., 2016; Liu et al., 2024) and the Hedgehog pathway intermediate Smoothened (Khaliullina et al., 2015). The *Drosophila melanogaster* genome does not encode homologs of the CB1 and CB2 receptors (McPartland et al., 2001; McPartland et al., 2006). However, previous studies have already shown effects of cannabis extracts and synthetic cannabinoids on diverse aspects of *D. melanogaster* biology (Ahn et al., 2021; Candib et al., 2024; He, Tan, et al., 2021; Kawasaki et al., 2025). Therefore, fruit flies could serve as an excellent experimental model to dissect the genetic mechanisms of cannabinoid function via any of the CB1/2-independent targets listed above, for all of which there exist *Drosophila* homologs. For instance, Jacobs and Sehgal showed that a metabolite derived from the endocannabinoid anandamide protects flies against seizures, and used a targeted genetic screen to determine that this effect was at least partially mediated by the TRP channel *water witch* (Jacobs & Sehgal, 2020).

Given that both alcohol and cannabis use are associated with a significant risk of developing into corresponding use disorders and addiction, it is not surprising that there has been a long-standing interest in understanding their potential interactions at both the neuropharmacological and molecular level. Much of the previous work in this area has focused on the role of endocannabinoids and CB1-dependent signaling on sensitivity and tolerance development to ethanol (reviewed in Pava & Woodward, 2012), with relatively less known about the potential role of non-canonical cannabinoid signaling on an organism’s response to alcohol. Of note, exposure to CBD and other non-psychoactive cannabinoids has been shown to attenuate behaviors related to ethanol tolerance and addiction in rodent models (reviewed in Turna et al., 2019), and diverse clinical trials have been designed to explore the use of cannabidiol and related substances for the treatment of substance use disorders (reviewed in Morel et al., 2021).

Foundational work by Scholz and colleagues demonstrated that flies can develop rapid functional tolerance to ethanol (RFTE; Scholz et al., 2000). Briefly, flies that have been pre-exposed to intoxicating doses of ethanol will show decreased sensitivity to a second ethanol exposure shortly after (hence, ‘rapid’), even when the internal ethanol concentration after the second exposure is comparable to that achieved after the first (hence, ‘functional’). Since this seminal discovery, *Drosophila* has served as an excellent model to study the neuronal, molecular and genetic mechanisms behind the development of RFTE (for reviews highlighting different advances in the field see Chvilicek et al., 2020; Engel et al., 2019 and Park et al., 2017). These previous studies have not only led to a rich theoretical framework for future studies, but also to the development of simple and scalable behavioral assays that can be easily implemented in the context of a Course-based Undergraduate Research Experience (CURE) class (e.g., Bhandari et al., 2009 and Sandhu et al., 2015). Therefore, we decided to explore whether long-term exposure to dietary phytocannabinoids might affect the ability of flies to develop rapid functional ethanol tolerance through a 300-level CURE offered at our institution. The work presented here was inspired by a previous study by He and colleagues, who reported that young males exposed to dietary cannabinoids for 2 and 4 days did not significantly affect their baseline sensitivity to ethanol, nor their ability to develop RFTE (He, Ng, et al., 2021). In our study, we decided to explore if exposing female flies to food containing cannabinoids for a longer period (14 days) might affect their ability to develop RFTE.

When adult females were maintained on a CBD diet for 14 days, their baseline sensitivity to ethanol sedation was comparable to that of flies on a control diet (CBD_Nv vs. CT_N; Fig. 1f). As expected, control flies pre-exposed to an intoxicating dose of ethanol showed a significant increase in ST50 (CT_N vs. CT_Pre; Fig. 1f), reflecting their ability to develop RFTE. Surprisingly, flies on a 14-days CBD diet did not show a significant increase in ST50 following a pre-exposure to ethanol (CBD_N vs. CBD_Pre; Fig. 1f), indicating that they had lost such ability. We also tested the mean climbing percent of flies across groups at their corresponding ST50; i.e., how high, on average, the remaining 50% of unsedated flies in a group could climb at the ST50. This comparison revealed no significant differences across all groups (Fig. 1g), suggesting that prior intoxications and pre-exposure to dietary CBD affected the onset of sedation and the ability of flies to develop RFTE, but not the climbing capacity of those that remain unsedated.

Lastly, we sought to determine if flies exposed to CBD during larval development, instead of as adults, would also lose their ability to develop RFTE. Adult parents were kept for 4 days to lay eggs in bottles containing regular food (CT) or supplemented with CBD oil (CBD), and then removed. Their progeny was then left to develop into adulthood in either diet, and adults were tested 1-4 days after eclosion for their ability to develop RFTE. We did not observe any noticeable differences in larval size, eclosion rates or the size and overall behavior of adults under either diet. In addition, there was no difference in the baseline ethanol sensitivity of flies that developed in regular vs. CBD food (CT_N vs. CBD_N; Fig. 1h), and flies exposed to CBD during larval development retained their ability to develop RTFE, as shown by a significant increase in ST50 following pre-exposure to ethanol in both groups and a non-significant interaction between diet and pre-exposure to ethanol in a 2-way ANOVA (Fig. 1h).

Our findings are inconsistent with a report by He et al., who showed that pre-exposure to cannabinoids did not significantly affect the ability of adult flies to develop RFTE (He, Ng, et al., 2021). However, there exist two important differences between our studies that may warrant deeper exploration: i) the sex and genetic strain of the flies used ( *Canton S* males in He, Ng et al., 2021 vs. *w ^1118^* females in our study); ii) the time of exposure to cannabinoids (2-4 days for He, Ng et al., 2021 vs. 14-days in this study).

Given that CBD acts as a natural antagonist of CB1R, and CB1R antagonists have been shown to reduce the self-administration and appetitive value of ethanol in rodent models (Arnone et al., 1997; Economidou et al., 2006), as well as inhibit the development of rapid tolerance to ethanol in rats (Lemos et al., 2007), CBD had long been viewed as a promising pharmacotherapy candidate to treat alcohol used disorders (AUD). Accordingly, CBD treatment was shown to inhibit the context-dependent relapse into alcohol seeking in rats with a history of alcohol consumption (Gonzalez-Cuevas et al., 2018), and their ability to develop tolerance to sedation by alcohol following re-exposure (Szulc et al., 2023). Not surprisingly, most previous work has focused on the potential role of CB1/2R-dependent signaling in the cross-talk between ethanol and CBD. Notably, Viudez-Martinez and colleagues showed that a 5HT1A serotonin receptor antagonist could block the reduction in alcohol self-administration triggered by the combined treatment with CBD and naltrexone (Viudez-Martínez et al., 2018), establishing a precedent for the potential involvement of non-canonical cannabinoid signaling in the cross-talk between CBD and alcohol.

It should be noted that we cannot rule out indirect effects of CBD on a fly’s response to ethanol. For instance, exposure of adults to a CBD diet for more than two days was shown to slightly but significantly reduce appetite in flies (He, Tan et. al, 2021). If our longer-term exposure to CBD effectively reduced their appetite even further, the observed effect of CBD could potentially be explained by a hidden connection between reduced nutrition and a diminished capacity for RFTE. Similar hypotheses could be explored for a diverse range of physiological axes, making this experimental paradigm particularly appealing in the context of a CURE class, in which students without prior Drosophila experience can still formulate and test a wide range of their own mechanistic hypotheses (e.g. effect of sex, age, genetic background, CBD and ethanol dosing, temperature, co-treatments, social stress, etc).

More broadly, and whether through more direct or indirect mechanisms, the findings reported here suggest that *Drosophila* could be added as a powerful experimental model to further explore the role of non-canonical pathways in the development of alcohol tolerance, and their potential use as new therapeutic targets to treat AUD.

## Methods

Two vials containing 45 females and 15 males from a standard *w ^1118^* stock were maintained for 14 days in 2mL of control or CBD food, and flipped every 2-3 days (see Reagents for details). CBD food was prepared by dissolving 120mg of the original oil in 1mL ethanol (used as a carrier), and diluted 1/230 into molten fly food at or below 65°C during preparation (for a 0.52mg/mL, or approximately 1.65mM final concentration). On day 15, flies were pooled and females from each group (CT or CBD) were re-sorted into regular food vials (40 flies/vial) and left to recover overnight.

Ethanol sedation cages were prepared once by perforating 4 small (~2-3mm) equidistant holes on the back side of a 10cm-square plastic Petri dish, approximately 5mm from the edge and adding a strip of white tape on the opposite side, to aid in distinguishing between sedated and non-sedated flies (Fig. 1a). Before each sedation assay, a rolled-up 10x10cm medical gauze is taped to the top, covering the pinholes (Fig. 1b).

On the morning of day 16, both vials from each group were randomly labeled as ‘naïve’ (Nv) or ‘pre-exposed’ (Pre), thus generating four experimental groups: CT_Nv; CT_Pre; CBD_Nv and CBD_Pre. Flies in both pre-exposed groups were transferred by tapping into empty vials capped with half-plugs soaked in 1mL of pure ethanol. Flies were exposed to ethanol vapors for 10 minutes and transferred back into regular food vials by tapping and left to recover for 4hrs (keeping the vials horizontal to prevent the sticking of sedated flies to the food surface).

In our CURE class, students transferred the flies to empty vials and “ice-nesthetized” them by incubating the vials on ice for 10 minutes. Ice-nesthetized flies were transferred by gentle tapping into sedation cages, which were immediately sealed with parafilm. Flies were left to recover from ice-nesthesia for 15 minutes at room temperature. After the 15min recovery, 4mL of ethanol were pipetted onto the gauze rod at the top of the cage through the pinholes in the back (1mL/pinhole, to facilitate even distribution). The pinholes were then taped over, the cages were placed in front of a white background and a timer was started. Students took frontal pictures of the cage every 30 seconds (Fig. 1c), until all flies were observed at the bottom of the cage in two consecutive pictures. It should be noted that the sedation cages were not tapped at regular intervals before taking the pictures. Therefore, our assay did not measure sedation as a reduction in the evoked negative gravotaxis reflex triggered by startling in flies, but on a more general loss of motor control and adhesion to vertical surfaces.

Pictures were opened as an image sequence using ImageJ/Fiji, and the multi-point selection tool was used to “spot” all flies, except those visibly laying on their side or backs at the bottom of the cage. The Analyze>Measure function in ImageJ/Fiji was then used to generate a table with X/Y coordinates of all the spotted flies, which were copy/pasted into an MS Excel spreadsheet. Using the MIN and MAX formulas across all time points, the relative position of each fly was re-calculated as (Y ^val^ -Y ^MIN^ )/(Y ^MAX^ -Y ^MIN^ )*100, to convert all Y-climb values to a 0-100 scale and standardize measurements across groups. The re-scaled Y-climb values were copied to a cross-tab table (with columns for time points and rows for Y-climb values of spotted flies). Each column was then used to calculate a mean Y-climb for each time point, and the COUNT function was used to calculate the percent of active flies at each time point (as #spotted flies/total flies in the cage*100), both of which were used to generate line plots of mean Y-climb or %Act vs. time (e.g., Fig. 1d). A linear regression was then used to calculate ST50 values for each group (i.e. the time at which 50% of the flies were sedated), by solving (50-Y ^intercept^ ) / slope, based on the corresponding line equation. The calculated ST50 were treated as biological replicates across groups (Fig. 1e), and ANOVA was used to compare sedation rates across treatments. The climbing capacity for a sample at ST50 (Fig. 1g), was calculated by solving %Y-climb ^ST50^ = slope * ST50 + Y ^intercept^ , based on a linear regression of %Y-climb values over time.

Each experiment was repeated via three independent trials throughout the semester, and only the results consistent across the three trials are reported here. Results were analyzed via parametric or non-parametric 1- and 2-way ANOVAs (followed by pairwise post-hoc comparisons when applicable), using Prism (GraphPad) and the open-source statistical package JASP (ver 0.95.0; https://jasp-stats.org/). &nbsp;Note that only the outcomes of more relevant pairwise comparisons are depicted by brackets in the graphs: i.e., Nv vs. Pre for each diet (reflective of effects on RFTE), and Nv vs. Nv between diets (reflective of effects on baseline sensitivity to ethanol).

## Reagents

**Table:** 

Reagent	Description	Source	Pdt/Cat #
Fly strain	* w ^1118^ ; + ; + ; + *	BDSC	RRID:BDSC_3605
Control food	Drosophila Agar = 1% w/vCornmeal = 5.9% w/vMolasses = 5.0% v/vYeast Extract = 2.4% w/vTegosept = 0.13% w/vPropionic acid = 542mMPhosphoric acid = 2.95mM	GeneseeScientificFisherScientific	66-10362-10062-11762-10620-258AC149300010A242-4
29:1 CBD:THC food	Control food + Full spectrum Cannabis Oil (724mg/mL CBD mix [cannabidiol, cannabigerol, cannabinol, cannabichromene, cannabidoilic acid] + 23mg/mL THC)	Proof Operations, Inc	F0103-111523
Square TC plates	10cm Square Petri Dish	Amazon/BIPEE	B01DBBBZO4
Square gauze pads	10cm Square 4-ply wipes	Amazon/Perfect Stix	B0791Y5WS6
Vials & Plugs	Narrow PS vials + Flugs	Genesee Scientific	32-109BF
Ethanol	Standard 200-proof ethanol	Pharmco	111000200
